# Mindfulness as Moderator Against Emotional Exhaustion Due to Online Teaching During COVID-19 Pandemic: An Investigation Using Job Demands-Resources Model and Conservation of Resource Theory

**DOI:** 10.3389/fpsyg.2021.781804

**Published:** 2021-12-15

**Authors:** Chuan-Chung Hsieh, Sophia Shi-Huei Ho, Hui-Chieh Li, Jyun-Kai Liang

**Affiliations:** ^1^Department of Education and Learning Technology, National Tsing Hua University, Hsinchu, Taiwan; ^2^Institute of Educational Administration and Evaluation, University of Taipei, Taipei, Taiwan; ^3^Center for Teacher Education, National Tsing Hua University, Hsinchu, Taiwan; ^4^Department of Applied Psychology, Hsuan Chuang University, Hsinchu, Taiwan

**Keywords:** mindfulness in teaching, online teaching, emotional exhaustion, JD-R model, COR theory

## Abstract

On the basis of the Conservation of Resource (COR) theory and using the Job Demands-Resources (JD-R) model, this study examines the relationships among job demands and job resources of online teaching (JD-OT and JR-OT), perceived instructional efficacy of OT (PIE-OT), mindfulness in teaching (MiT), and emotional exhaustion (EE) to understand the psychological stress experienced by teachers engaged in OT and how mindfulness has moderating effects on relieving anxiety and preventing burnout. A total of 476 teachers with OT experience completed online a self-report survey with items adapted from related scales. The hypotheses were validated using structural equation modeling. Causal relationships were assessed using path analysis, and multi-group analysis was performed to examine the moderating effect of MiT. JD-OT has significant and negative impact on PIE-OT, JR-OT has significant and positive impact on PIE-OT, and PIE-OT has significant and negative impact on EE. Moreover, PIE-OT mediates the positive relationship of JD-OT with EE and the negative relationship of JR-OT with EE. The moderating role of MiT in the relationship of JD-OT and JR-OT with PIE-OT was also validated. In OT work environments, teachers have great need and desire for JR, which can have a positive impact on PIE. Mindfulness training contributes to improving OT efficacy and reducing EE. Enhancing teachers’ MiT enables them to deal with demands from work and their superiors and motivates them to respond with ease to the stressful external environment.

## Introduction

The outbreak of the COVID-19 has significant impact on people from all walks of life and all sectors in society. In the education sector, schools are shut, and teachers and students are forced out of the classrooms. These drastic changes have resulted in a distinctive and unprecedented rise in remote or e-learning and online teaching (OT) worldwide. In Asia, Taiwan has been an outlier with face-to-face classes maintained during the pandemic till May 15, 2021. Following triple-digit numbers of domestically transmitted COVID-19 cases, schools at all levels were closed. On-site classes were suspended but teaching and learning continued through online platforms. Such transition though necessary is not easy; and the speedy response required under the pandemic does not allow changes to be implemented in small or steady steps.

To teachers, their nature of work transforms radically almost overnight. Not only do they switch from school- to home-based, the lessons, assignments, and assessments have to be tailored to the online format. This sudden shift of teaching delivery mode poses challenges to teachers for adapting and surviving in the new normal. In addition, it is expected of them to support students’ academic development and psychological well-being throughout this transition. Teachers experienced considerable stress as a result of the COVID-19 pandemic, which was related to poorer mental health, coping, and teaching (Baker et al., 2021). In view of the significant role of teachers in knowledge dissemination, it is important to ensure sufficient support and prompt assistance be rendered to facilitate their managing the drastic changes (Mishra et al., 2020). Hence, an in-depth understanding of the challenges and predisposing factors of their stress and anxiety as well as how to help teachers overcome these problems would be of need and value.

From diverse perspectives, a plethora of studies have examined the impact of COVID-19 lockdown on home education (Cahoon et al., 2021), social life and mental health of students (Chaturvedi et al., 2021) competences of parents, children, and teachers (Öçal et al., 2021), and student academic performance (Iglesias-Pradas et al., 2021). With face-to-face teaching replaced by remote instruction, new approaches to curriculum design (Yilmaz et al., 2021), OT activity design and implementation (Wu, 2021), and teachers’ technology acceptance (Hong et al., 2021) have been studied.

Teaching is generally considered a highly stressful profession (Harmsen et al., 2018), and the COVID-19 crisis has made things even worse (Kumawat, 2020). Online teaching had a negative psychological impact on teachers, causing them to feel anxious, stressed, and depressed (Cheng and Lam, 2021; Ozamiz-Etxebarria et al., 2021). Pressley (2021a) found that the antecedents to teacher burnout-stress were COVID-19 anxiety, current teaching anxiety, anxiety communicating with parents, and lack of administrative support. Ma et al. (2021a) reported that the lack of experience in OT caused teachers to have low perceived self-efficacy.

Burnout in teachers has been broadly investigated, but limited studies have investigated burnout in teachers during a pandemic (Sokal et al., 2020). In particular, because the environment changes so rapidly, the resources to cope with the changes are often not ready in time. Schools require teachers to teach online while still maintaining as much as possible the effectiveness of face-to-face teaching. Teachers need to spend extra time to make themselves familiar with the online teaching environment, designing methods to interest students in the teaching content and keep them learning about it (Scull et al., 2020). This creates a great deal of psychological pressure on teachers. Prior organizational studies have found that job demands lead to constant psychological overtaxing and exhaustion (Bakker et al., 2008) while lack of job resources not only predicts burnout but also poor engagement (Schaufeli and Bakker, 2004). In view of the core role of teachers in education, it is important to assess the psychological impact of OT, necessitated by COVID-19, on them and provide them with prompt support and assistance to reduce work pressure and improve teaching effectiveness (Mishra et al., 2020). In particular, OT and e-learning would be the modus operandi till the pandemic is over and may even remain to some extent in its aftermath.

In recent years, mindfulness has been gaining recognition in the field of psychotherapy and is considered an effective intervention for individuals facing life pressure, depression, and anxiety (Hofmann et al., 2010). Kabat-Zinn (2003) defined mindfulness as “the awareness that emerges through paying attention on purpose, in the present moment, non-judgmentally to the unfolding of experience moment by moment.” Mindfulness has been found to influence teachers’ occupational health and well-being through reducing depression, anxiety and perceived stress (Braun et al., 2019; Ma et al., 2021b). Every individual has varied levels of mindfulness in different situations and time. Brown and Ryan (2003) referred to an individual’s capacity of paying and maintaining attention to present moment experiences with an open and non-judgmental attitude as trait mindfulness or dispositional mindfulness. Moreover, individuals with higher trait mindfulness tend to have stronger self-regulated behavior and positive emotional states (Brown and Ryan, 2003). Trait mindfulness has also been demonstrated to have positive downstream effects on workplace functioning (Dane, 2011; Glomb et al., 2011).

Enhanced awareness and de-automatized response emphasized in mindfulness enable teachers to be more self-conscious and at the same time to perceive better their students, thus facilitating more flexible management and adjustment. Moreover, contributive to teacher-student interactions are characteristics of mindfulness including focus on the present, openness, non-judgmental, and acceptance, which would help teachers be more understanding and accommodating, perceptive and supportive. Combining teacher intrapersonal and interpersonal mindfulness, Frank et al. (2016) put forward the Mindfulness in Teaching Scale for predicting burnout and social-emotional self-efficacy. Among kindergarten teachers, mindfulness was found to be effective in reducing burnout (Ma et al., 2021b). Hence, this study aims to explore whether mindfulness in teaching would also help relieve anxiety and emotional exhaustion attributed to the sudden but persisting switch to OT.

As mentioned above, an in-depth understanding of the self-regulation mechanism of teachers confronting considerable stress because of the COVID-19 pandemic is eagerly anticipated. This study, therefore, examines the relationships among job demands and job resources of OT, perceived instructional efficacy of OT, mindfulness in teaching and emotional exhaustion to understand the psychological stress experienced by teachers engaged in OT and how mindfulness has moderating effects on relieving anxiety and preventing burnout based on the Conservation of Resource theory and using the Job Demands-Resources model.

The remainder of this paper is organized as follows. The related theory and model are first reviewed as the basis of the hypotheses thus proposed. Then the measurement instruments for the different constructs are introduced. Details on data collection and processing as well as results obtained are described. The proposed hypotheses are then verified using structural equation modeling. Finally, research discussion and limitations are presented.

## Theoretical Background and Research Hypotheses

### Theoretical Background

#### Conservation of Resources Theory

Proposed by Hobfoll (1989), the Conservation of Resources theory (COR) is a good foundation for understanding stress and its relationship with the supply and demand of resources for individuals and society. Resources, precious but limited, are spent in daily life and interactions. When faced with loss and lack of resources, psychological stress occurs. In response to such stress, individuals on one hand preserve their current resources and on the other hand pursue new ones. Whether successful or not, their attempts would be resource-depleting and emotionally taxing. Job burnout is a work-related stress that arises under excessive workload and pressure and leads to energy and emotional exhaustion as well as reduced performance. According to the COR, burnout at work occurs as a result of perceived or actual loss of energy under heavy job demands. For teachers, extra workload due to the new teaching delivery mode is energy-draining. In the absence of support or relief, their persistent efforts will eventually lead to job burnout.

The literature contains abundant studies on job burnout of teachers (Byrne, 1991; Burke and Greenglass, 1995; Dorman, 2003), and emotional exhaustion has been found to be the most significant aspect and impact of job burnout (Shirom, 1989; Taris et al., 2005). Students would not find it pleasant or fruitful to interact with a lethargic teacher who feels frustrated and whose enthusiasm is drained under work pressure. Moreover, the effectiveness of instruction would be undermined when teachers are emotionally exhausted. In the meta-analysis of Lee and Ashforth (1996), both demand and resource correlate showed strong relationships with emotional exhaustion. In this study, emotional exhaustion is taken as the outcome variable.

#### Job Demands-Resources Model

The job demands-resources model (JD-R), proposed by Demerouti et al. (2001), is the COR theory put to actual practice at the workplace and identifies a wide array of significant links between both positive and negative aspects of work environments. The working condition of every occupation has two components: job demands (JD) and job resources (JR). JD refer to those physical, psychological, social, or organizational aspects of the job that require sustained physical and/or psychological (cognitive and emotional) effort or skills and are therefore associated with certain physiological and/or psychological costs. Examples are work pressure, an unfavorable physical environment, and emotionally demanding interactions with clients. Although JD are not necessarily negative, they may turn into job stressors when meeting those demands requires high effort or competences that the employee is not equipped with. On the other hand, JR refer to those physical, psychological, social, or organizational aspects of the job that are instrumental to enhancing work engagement and organizational commitment as well as to achieving work goals and better performance (Bakker et al., 2003). Examples are job autonomy, role clarity, collegial, and/or supervisory support. Hence, according to the JD-R model, present at all work environments are contrasting processes: exhausting/destructive and stimulating/constructive.

Using the JD-R approach, Bakker and Demerouti (2007) explored the impact of JD and JR on employees’ well-being and performance. A recent study of Collie (2021) examined the relationship between principal leadership and teachers’ stress and emotional exhaustion during the pandemic also using the JD-R model. OT necessitated by COVID-19 poses heavy a workload that demands teachers to make extra concentrated efforts as well as frequent interaction with students and parents, thus incurring psychological stress. With increasing JD of OT (JD-OT), internal resources are further exhausted. Worse still, if JR including work autonomy, school, and coworkers’ support are lacking, OT would be even more energy-consuming. Continuous draining of energy and resources will result in burnout at high psychological cost (Lee and Ashforth, 1996).

#### Trait or Dispositional Mindfulness

The relationships between JD-OT and the subsequent psychological processes and costs are affected by individual differences of teachers. Teaching-related traits, such as self-confidence and enthusiasm, are an individual’s internal resources whose depletion showed individual variation. That is to say, positive traits of an individual can help resist stress and slow down resource depletion (Hobfoll, 1989). The more positive traits a teacher has, the less resources are consumed to meet the JD and the less severe the emotional exhaustion.

In this study, mindfulness is taken as an individual’s positive trait and resource. When applied to an educational setting, mindfulness can regulate the psychological process and ameliorate the psychological costs of teachers with JD and (lack of) JR of OT. According to Frank et al. (2016), teachers engaged in mindfulness should have dual focus, intrapersonal mindfulness for dealing with their own mental health and interpersonal mindfulness for supporting responsive teaching behavior beneficial to students. Hence, mindfulness in teaching (MiT) should on one hand reduce emotional exhaustion (EE) and prevent burnout and on the other hand improve perceived instructional efficacy (PIE) and enhance student performance.

### Research Hypotheses

**Figure 1:**
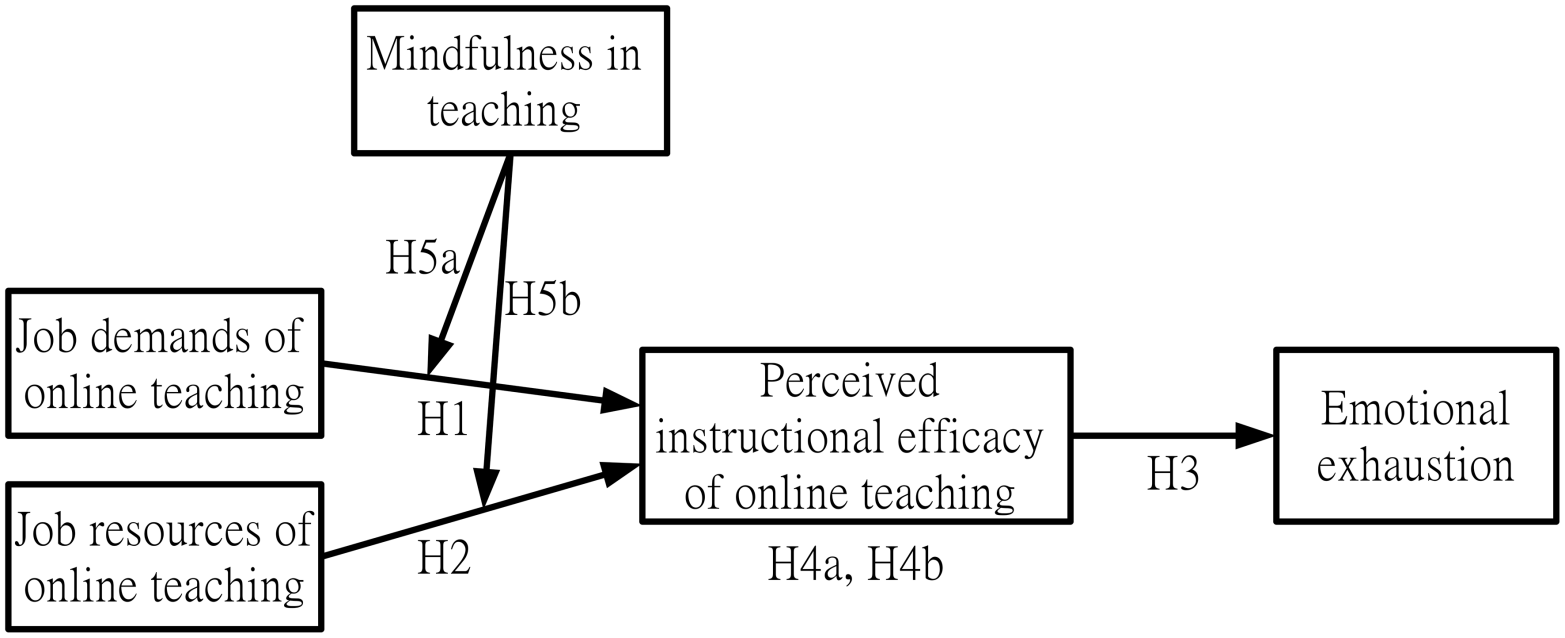
Research framework.

Figure 1 shows the research framework of this study. Details of each research variable as well as their hypothesized relationships are presented in the following.

#### Relationship of JD-OT and JR-OT With PIE-OT

JD-OT faced by teachers include work pressure, cognitive load, and emotional requirements while JR-OT include work autonomy, social support from colleagues, school guidance, performance feedback, and professional development or career opportunities. Kinman et al. (2011) reported evidence that workplace social support lessens the negative impact of JD on EE and job satisfaction.

Self-efficacy, a concept put forward by Bandura (1977), refers to an individual’s judgment of how well he/she can produce actions to attain specific performance required. Teacher self-efficacy thus denotes a teacher’s belief in his/her ability to successfully cope with tasks, obligations, and challenges related to his/her professional role (Caprara et al., 2006). Dussault et al. (2004) investigated the relationship between teachers’ IE and their efficacy toward integration of technologies in the classroom. Pressley (2021b) showed that teachers teaching virtually had the lowest IE scores compared with teachers teaching in a hybrid or all in-person model. In this study, PIE-OT is taken as a research variable, which reflects a teacher’s beliefs in his/her capacity in executing OT, using auxiliary media, and designing new instruction materials for e-learning.

In the study of Skaalvik and Skaalvik (2019) on teacher efficacy, JD in the teacher profession identified from prior research include time pressure, discipline problems, conflicts with colleagues, lack of administrative support, and value conflicts. Their earlier study (Skaalvik and Skaalvik, 2010) on the impact of perceived JD and JR among teachers found time pressure to be the strongest predictor of EE and positive association of autonomy with self-perceived accomplishment. In addition, workplace social support can lead to greater organizational commitment and dedication (Bakker et al., 2003) while a supportive school climate has a positive impact on teacher self-efficacy (Aldridge and Fraser, 2016). The above findings reveal the significant influence of JD and JR on teachers’ perceived self-efficacy. According to the COR, the heavier the JD-OT, the greater the physical and psychological efforts are required, and the more personal resources are consumed, resulting in low PIE-OT. In view of this perspective, the following hypotheses are proposed.

*H1: JD-OT is inversely and negatively related to PIE-OT.*

*H2: JR-OT is linearly and positively related to PIE-OT.*

#### Relationship of PIE-OT With EE

As a stress component, EE refers to feelings of being emotionally overextended and depleted of one’s emotional resources. Signs of emotional exhaustion include fatigue, debilitation, loss of energy and wearing out (Schwarzer and Hallum, 2008). Besides contributing to burnout, emotional exhaustion should be of concern due to its association with impairment of overall job performance, well-being, and health (Ducharme et al., 2007). Teachers with low perceived self-efficacy are under great job stress and are likely to become emotionally exhausted (Schwarzer and Hallum, 2008). According to the COR, teachers spend more time and effort to deal with JD-OT, thus exhausting their personal and internal resources. Persistent challenging demands in the absence of support will erode teachers’ initial passion and enthusiasm, vitality and creativity, causing them to perceive themselves to be instructional inefficacious and eventually become emotionally exhausted. In view of this perspective, the following hypothesis is proposed.

*H3: PIE-OT is strongly and negatively related to EE.*

#### Pie-OT as Mediator

JD are primarily related to the exhaustion component of burnout, whereas lack of JR are primarily related to disengagement (Demerouti et al., 2001). The JD-R model predicts that JD increase and JR decrease EE in employees (Ruysseveldt et al., 2011). Using the JD-R model, Huang et al. (2019) observed mediating effects of teacher self-efficacy on the relationship between the emotional JD of teaching and well-being of primary school teachers in Hong Kong. Rabaglietti et al. (2021) found partial mediation effect of self-efficacy on the relationship between difficulties in organizing distance learning during the pandemic and perceived stress among teachers from European countries. Taken together, these results evidenced the important mediating role of self-efficacy on both positive and negative feelings of teachers in the teaching environment. According to the COR, the amount of JD and JR would determine the amount of physical and psychological efforts required and the extent of resource depletion, which in turn affect the severity of EE. In this study, PIE-OT is taken as the outcome of efforts paid and resources consumed, both of which are strongly related to EE. In view of this, the following hypotheses are proposed.

*H4a: PIE-OT mediates the relationship between JD-OT and EE.*

*H4b: PIE-OT mediates the relationship between JR-OT and EE.*

#### Mindfulness in Teaching as Moderator

Considered as a positive personality trait (Marzabadi et al., 2021), mindfulness practiced by teachers can help them perceive their own feelings and thoughts non-judgmentally in face of JD. With them focusing on their teaching tasks would reduce the negative impact of JD and lessen their physical and psychological burden. In other words, MiT serves as a buffer against energy-draining JD, decreases resource consumption, and prevents emotional fatigue. Conversely, teachers without this positive trait will have no shield against the pressing and taxing JD, resulting in greater physical and psychological efforts paid, which may undermine PIE. In addition, according to the COR, positive traits can also be regarded as personal resources that can be invested in the teaching task to reduce the negative impact of physical and psychological exhaustion and low PIE. Furthermore, MiT helps replenish JR under exhaustion, thus preventing burnout.

Past studies have explored the moderator role of mindfulness. When exploring the relation between income and life satisfaction, Sugiura and Sugiura (2018) reported individual differences in mindfulness as a possible reliable factor to enhance psychological well-being. In the field of clinical psychology, Zhong et al. (2020) found significant association between perceived stress and psychological symptoms in patients with low dispositional mindfulness. A randomized controlled trial conducted by Shapiro et al. (2011) showed that there is likely a moderating relationship between trait mindfulness and stress once trait mindfulness is added as a covariate. Nevertheless, the moderating effect of MiT when applied to an online work environment has not been proven (Frank et al., 2016). In view of these findings, the following hypotheses are proposed.

*H5a: MiT moderates the relationship between JD-OT and PIE-OT.*

*H5b: MiT moderates the relationship between JR-OT and PIE-OT.*

## Methodology

### Instruments

This study collected survey data using a structured questionnaire made up of standardized scales for measuring the different research variables. These well-developed scales are adapted and modified for the online teaching mode. For them to be administered to respondents in Taiwan, this study applied the back-translation technique (Brislin, 1980) to come up with a version in Traditional Chinese specially catered for online teaching situations. Respondents were asked to indicate their agreement to the statement in the questionnaire using a five-point Likert scale (1 indicates strongly disagree; 5, strongly agree). The following describes the scales used for each research construct.

#### Mit Subscale

This study adopted 14 items from the Mindfulness in Teaching Scale developed by Frank et al. (2016). A sample item is: “I am aware of how my moods affect the way I treat my students.” Among them, nine items, all reverse-coded, measure intrapersonal mindfulness and five items measure interpersonal mindfulness. Cronbach’s α for this 14-item subscale was 0.879.

#### JD-OT and JR-OT Subscale

This study used 29 items from the Job Demands-Resources Questionnaire developed by Bakker (2014). Related to JD were 4 items on workload (such as “I have too much work to do”), 4 items on cognitive demands (such as “My work demands enhanced care or precision”), and 4 items on emotional demands (such as “I face emotionally charged situations in work”). Cronbach’s α for these 12 items was 0.889. Under the dimension of JR were 3 items on autonomy (such as “I can decide myself how I execute my work”), 3 items on social support (such as “I can ask my colleagues for help if necessary”), 3 items on feedback (such as “I receive sufficient information about the results of my work”), 5 items on coaching (such as “My supervisor is friendly and open to me”) and 3 items on opportunities for development (such as “My work offers me the opportunity to learn new things”). Cronbach’s α for these 17 items was 0.864.

#### Pie-OT Subscale

Four items were extracted from the Chinese version of the teacher self-efficacy scale developed by Sun (2003) to measure PIE. A sample item is: “No matter how difficult the material is, I can still express it clearly to the students.” Cronbach’s α for this 4-item subscale was 0.710.

#### EE Subscale

Three items were adapted from the Shirom-Melamed Burnout Measure developed by Shirom (1989) to assess EE. A sample item is: “I feel I am unable to be sensitive to the needs of students.” Cronbach’s α for this 3-item subscale was 0.842.

### Participants

An online survey study was conducted for 3 weeks in mid-June, 2021 in Taiwan. Participants were recruited through snowball sampling and convenience sampling. The URL of the online survey was sent to teachers’ communities through social networking platforms including Facebook and Line inviting their participation. The survey sample comprised elementary school, junior high school, senior high school, and university teachers with OT experience. A total of 476 participants completed the online survey. There were more male (72.7%) than female teacher respondents (27.3%). Among the respondents, 64.5% were elementary school teachers 16.8% were junior high school teachers, 14.5% were senior high school teachers, and 4.2% were university teachers; 33.6% had been teaching for more than 21 years; 22.7% for 16–20 years, and 43.7% for less than 15 years; 27.9% had bachelor’s degree, 63.4% had master’s degree, and 8.6% had doctoral degree.

### Analytical Method

The analysis in this study followed the two-step rule put forward by Bollen (1989). The first step examined the goodness-of-fit of each latent variable using confirmatory factor analysis (CFA), while the second step assessed the causal relationships among the latent variables using path analysis. The hypotheses were validated using structural equation modeling (SEM). The maximum likelihood (ML) was estimated using AMOS 22.0 to evaluate the goodness-of-fit of the hypotheses for the empirical data. A measurement model typically considered as having a good fit to the data has *x*^2^/df ranging from 2 to 5, goodness-of-fit statistic (GFI) value of 0.9 or above, comparative fit index (CFI) value of 0.9 or above and a root mean square error of approximation (RMSEA) value of 0.08 or less (Kline, 2005).

To determine the moderating effect of MiT, the respondents were divided into three groups according to their MiT scores, with the top one-third categorized as high (H)-MiT and the bottom one-third as low (L)-MiT. Then, multi-group analysis (MGA) was performed on the H-MiT and L-MiT groups. First, the standard model (unrestricted model), which allows different path coefficients between groups, was applied, thus yielding a chi-square value ( $\chi_{\mathrm{unre}}^{2}$ ). Then, the restricted model, which constrains all path coefficients of both groups to be equivalent, was applied, thus obtaining another chi-square value ( $\chi_{\mathrm{re}}^{2}$ ). The standard model and the restricted model are both nested models that can be statistically compared. A significantly lower Δ*χ*^2^ value for the standard model (*p* < 0.05) indicates a poorer fit of the restricted model. The hypothesis with the same path coefficients across groups is rejected, thus verifying the moderating effect between high and low groups (Hair et al., 2009).

## Empirical Results

### Relationships Between Variables

Table 1 lists the means, standard deviations, and correlations among the study variables. As can be seen, JD-OT was significantly and positively related to EE (*r* = 0.27, *p* < 0.005) but negatively related to PIE-OT (*r* = −0.26, *p* < 0.005); JR-OT was positively related to PIE-OT (*r* = 0.33, *p* < 0.005) but negatively related to EE (*r* = −0.22, *p* < 0.005); and PIE-OT was negatively related to EE (*r* = −0.46, *p* < 0.005).

**Table 1:** Means (M), standard deviations (SD), and correlations among study variables.

Variables	*M*	*SD*	1	2	3	4	5
1. MiT	3.82	0.47	0.61				
2. JD-OT	3.97	0.55	−0.15^**^	0.76			
3. JR-OT	3.72	0.48	0.21^**^	−0.05	0.74		
4. PIE-OT	3.29	0.64	0.41^**^	−0.26^**^	0.33^**^	0.57	
5. EE	2.46	0.87	−0.64^**^	0.27^**^	−0.22^**^	−0.46^**^	0.82

** *p <* 0.05.

Numbers in the diagonal rows are the square roots of the AVE.

### CFA Results, Convergent and Discriminant Validity

Results obtained from CFA revealed the measurement models having satisfactory goodness-of-fit. For job demands, the fit assessment results were *x*^2^/*df* = 2.297; GFI = 0.959; CFI = 0.976; RMSEA = 0.052. For job resources, the fit assessment results were *x*^2^/*df* = 4.004; GFI = 0.885; CFI = 0.905; RMSEA = 0.080. For teaching mindfulness, the fit assessment results were *x*^2^/*df* = 4.206; GFI = 0.930; CFI = 0.934; RMSEA = 0.080. Convergent validity of the scale used was also confirmed. First, all factor loadings of each construct exceeded 0.5 and were significant with value of p at 0.05. Second, the composite reliability (CR) of each latent variable exceeded 0.6, ranging from 0.65 to 0.95. Third, the average variance extracted (AVE) of each construct exceeded 0.5 (Fornell and Larcker, 1981; Bollen, 1989), ranging between 0.55 and 0.68, except for PIE-OT (AVE = 0.32). Taken together, these results indicate good convergent validity. Discriminant validity was verified by examining the square root of the AVE of each construct. According to the results presented in Table 1, the correlations among constructs are smaller than the square root of the AVE of each construct (Fornell and Larcker, 1981), indicating good discriminant validity.

### SEM Results

Table 2 summarizes the SEM results of hypothesis testing on direct effects and mediation effects. The fit assessment results were *x*^2^/*df* = 2.697; GFI = 0.831; CFI = 0.986; RMSEA = 0.060), indicating overall goodness-of-fit. As can be seen, H1, H2, and H3 are all supported, indicating direct effects of the research variables. That is, JD-OT has significant and negative impact on PIE-OT (H1, *β* = −0.23, *p* < 0.001), JR-OT has significant and positive impact on PIE-OT (H2, *β* = 0.75, *p* < 0.001), and PIE-OT has significant and negative impact on EE (H3, *β* = −0.53, *p* < 0.001). Moreover, H4a and H4b are both supported, evidenced by the absence of zero in the bootstrap 95% confidence intervals (CI; Shrout and Bolger, 2002), thus revealing significant mediation effects. In other words, PIE-OT mediates the positive relationship of JD-OT with EE (0.12, 95% CI [0.04, 0.16]) and the negative relationship of JR-OT with EE (−0.40, 95% CI [−0.37, −0.21]).

**Table 2:** Results of hypothesis testing on direct effects and indirect mediation effects.

Hypothesis	Path	*β*	*t*	95% CI	Results
H1	JD-OT → PIE-OT	−0.23	−3.96^***^	–	Supported
H2	JR-OT → PIE-OT	0.75	8.84^***^	–	Supported
H3	PIE-OT → EE	−0.53	−7.22^***^	–	Supported
H4a	JD-OT → PIE-OT → EE	0.12	–	[0.04, 0.16]	Supported
H4b	JR-OT → PIE-OT → EE	−0.40	–	[−0.37, −0.21]	Supported

*** *p <* 0.001.

### MGA Results

MGA results shown in Table 3 reveals moderating effect of MiT, both high and low levels, on the negative relationship of JD-OT with PIE-OT (−0.34 and − 0.14, respectively) and the positive relationship of JR-OT and PIE-OT (0.60 and 0.86, respectively). The differences in chi-square values of the two relationships above when assessed by standard and restricted models Δ*χ*^2^ are 6.58 and 5.37 with *p* = 0.010 and *p* = 0.020, respectively. With both *p*-values less than 0.05, the hypothesis that the path coefficients are the same across groups is rejected. In other words, H5a and H5b are supported, evidencing the moderator role of MiT in the relationship of JD-OT and JR-OT with PIE-OT.

**Table 3:** Moderating effect of mindfulness in teaching.

Compared with unrestricted model					
Path/Group	*β*	*SE*	*t*	*βs*	Δ*χ*^2^(*df* = 1)
JD-OT → PIE-OT					6.58 (*p* = 0.010)
L-MiT	−0.03	0.02	−1.46	−0.14	
H-MiT	−0.22	0.07	−3.13^*^	−0.34	
JR-OT → PIE-OT					5.37(*p* = 0.020)
L-MiT	0.16	0.05	2.99^*^	0.86	
H-MiT	0.39	0.08	4.65^***^	0.60	

L-MiT (*N* = 177), H-MiT (*N* = 169). *β* = unstandardized path coefficient. *β*s = standardized path coefficient. SE = standard error. Bootstrap samples = 1,000. Bias correction method: 95% CI of unstandardized coefficient.

* *p* < 0.01;

*** *p* < 0.001.

## Discussion and Conclusion

### Discussion

To the best of our knowledge, this study is the first conducted in Taiwan to examine the psychological process of EE among teachers facing a sudden shift to OT, the new teaching delivery mode necessitated by the COVID-19 pandemic when distance instruction replaces face-to-face teaching. On the basis of the COR and using JD-R model, this study explored the relationships of JD and JR with PIE and EE in an OT work environment to shed light on the psychological changes due to the sudden switch in teaching delivery mode necessitated by the COVID-19 pandemic. In particular, the moderating effect of MiT was examined for a better understanding on its application in the field of education.

Empirical data obtained from the online survey evidenced the impact of JD-OT and JR-OT on PIE-OT (H1 and H2, respectively). These results echoed previous findings on the negative effect of JD (Skaalvik and Skaalvik, 2019) as well as positive effect of JR and social support (Minghui et al., 2018; Skaalvik and Skaalvik, 2019) on teachers’ sense of self-efficacy. A recent study of Ma et al. (2021a) attributed low PIE to the teachers’ lack of OT experience and the school’s inadequate support. The negative impact of PIE-OT on EE (H3) evidenced by the empirical data was also consistent with prior results on negative relationship of PIE with EE (Skaalvik and Skaalvik, 2010) and job burnout due to low PIE (Skaalvik and Skaalvik, 2007).

Besides these direct impacts, indirect effects of JD-OT and JR-OT on EE with PIE as mediator (H4a and H4b, respectively) revealed by the empirical results were in agreement with the mediation effect of PIE previously reported (Huang et al., 2019; Rabaglietti et al., 2021). In summary, JD-OT undermines PIE but contribute to EE. Stress, physical efforts, and emotional load all cause low PIE, which indirectly leads to serious EE. On the contrary, JR-OT foster PIE but reduce EE. Work autonomy, social support, performance feedback, supervisory guidance, or development opportunities all promote PIE, which indirectly decreases EE. Of note is that the impact of JR-OT, both direct and indirect, is greater than JD-OT. This finding in an OT context echoes Ma et al.’s (2021a) study to point out that supportive school administration, a type of JR, is identified as one of the major associated factors to teaching self-efficacy. However, this finding is different from previous empirical results reported by Hakanen et al. (2006) and Skaalvik and Skaalvik (2019), showing greater positive and negative effects of JD compared with JR. In particular, Sokal et al. (2020) conclude most demands are most strongly correlated with the initial exhaustion stage of burnout during COVID-19 pandemic.

The COVID-19 pandemic and the consequent switch to OT have caused teachers to feel anxious, stressed, and depressed (Cheng and Lam, 2021; Ozamiz-Etxebarria et al., 2021). However, these studies have not explored the possible influence of OT work environment and PIE on EE of teachers. This research probed into this unexplored aspect and evidenced the mediating role of PIE in the relationship between OT work environment and EE of teachers. Faced with heavier work pressure from OT, greater cognitive and emotional demands, low work autonomy, few learning opportunities, and lack of social support, teachers need to make extra efforts to cope with the negative impact of high JD and low JR. All these diminish PIE, which increases the severity of EE.

While the moderating effect of MiT on the impact of JD-OT and JR-OT on PIE-OT (H5a and H5b, respectively) was evidenced by the empirical data, the magnitude of such effect was not as hypothesized. In comparison, the mean PIE score of the L-MiT group (3.02; SE = 0.55) was significantly (*p* < 0.001) lower than that of the H-MiT group (3.56; SE = 0.66), indicating that teachers with high MiT do consider themselves performing better at OT. For teachers with low MiT, JD-OT does have negative but insignificant impact on PIE-OT (−0.14) while the impact of JR-OT on PIE-OT is both positive and significant (0.86). L-MiT teachers show greater need of external resources and tend to look out for external support in face of extra demands. The basic assumption of COR is that individuals strive to acquire, maintain, cultivate and protect their cherished resources (Hobfoll et al., 2018). Hence, in face of pressed response and stressed requirement to enhance OT efficacy, the self-defense mechanism of L-MiT teachers is triggered, driving them to seek available resources for support while conserving their internal resources. This is consistent with one of the principles of COR. Hence JR-OT is of both need and importance to L-MiT teachers.

As for teachers with high MiT, JD-OT have a negative and significant impact on PIE-OT (−0.34) while the impact of JR-OT on PIE-OT is both positive and significant (0.60). In comparison, the negative effect of JD-OT on PIE-OT was greater in H-MiT; the positive effect of JD-OT on PIE-OT was also greater in L-MiT than in H-MiT. Such difference can be attributed to the proactive attitude of H-MiT teachers toward JD-OT even at the expense of their internal resources, which would decrease PIE. High mindfulness enables teachers face JD not only through pursuing external resources but also by exhausting internal resources, which can be compensated by the resources acquired. This process is similar to one of the core mechanisms of the ego depletion theory. An individual’s success of performing volitional activities is determined by the resources he/she possesses. The more sufficient the resources, the more likely he/she would succeed. Resources are exhausted temporarily and are replenished in due course (Baumeister et al., 1998).

### Practical Implications

Bakker and de Vries (2021) argued that job resources can fulfill psychological needs and buffer against the impact of JD on burnout. This study found that in the OT work environment, teachers have great need and desire for JR, which, if available and accessible, can have a positive impact on PIE. Hence, the education authority should offer guidance to schools at all levels in OT preparation and implementation and provide teachers with resources and platforms for digital learning. School administration and principals should take care of teachers’ psychological needs, help resolve OT-related problems, supply intangible resources, such as work autonomy, and establish communities for professional development so as to foster collegial support and enhance self-efficacy (Lee et al., 2011).

Mindfulness training contributes to improving OT efficacy and reducing EE. Enhancing teachers’ MiT enables them to deal with demands from work and their superiors and motivates them to respond with ease to the stressful external environment. Organizations should play the roles as supplier of resources and supporter against challenges and changes in the work environment. Schools can subsidize teachers to participate in mindfulness training courses, replenish resources for better performance in OT, and sustain teachers’ enthusiasm and energy.

Every country expects good-quality online teaching during the epidemic to reduce the impact on student learning. The effect of mindfulness on online teaching for teachers in Taiwan during the epidemic can be used as a reference for other countries. With the rapid spread of the epidemic, teachers needed to shift their teaching mode to OT in confusion. However, online teaching resources were not available to support them in time. Mindfulness allows teachers to sustain the effectiveness of online teaching, and it is less likely to lead to emotional exhaustion. The culture of Taiwanese society contains elements of Buddhism (Hwang, 2012), which is conducive to the promotion of mindfulness. However, past research on mindfulness has shown that it is still effective in different cultural society (e.g., Raphiphatthana and Jose, 2020). Therefore, we believe other countries are still worth trying to incorporate mindfulness into teacher training courses. It can increase the teacher’s ability to face the drastically changing environment.

### Limitations and Future Research

The survey in this study was conducted using a self-report questionnaire at a single point in time, which may cause common method variance (CMV). To minimize CMV, this study performed Harman’s one-factor test and applied exploratory factor analysis (EFA) to the 50 items in the questionnaire. The variance of the first variable introduced was 17.45%, which did not exceed 50%, implying that the CMV was not serious (Podsakoff et al., 2003). Moreover, the questionnaire designed in this study is concise and easy to understand, as suggested by Podsakoff et al. (2003). Some items are reverse-coded to reduce bias. Possible future research can be a longitudinal study with research constructs assessed at different time points, such as measuring JD-OT and JD-OT at the first time point and measuring MiT, PIE-OT, and EE at the second time point, to reduce the impact of CMV.

## Data Availability Statement

The raw data supporting the conclusions of this article will be made available by the authors, without undue reservation.

## Author Contributions

All authors contributed to the study conception and design. Material preparation, data collection, and analysis were performed by C-CH, SH, H-CL, and J-KL. The first draft of the manuscript was written by H-CL and J-KL. All authors commented on previous versions of the manuscript. All authors have read and approved the final manuscript.

## Funding

This study was partially supported by the grants from the Ministry of Science and Technology, Taiwan (MOST 109-2410-H-007-014-SSS).

## Conflict of Interest

The authors declare that the research was conducted in the absence of any commercial or financial relationships that could be construed as a potential conflict of interest.

## Publisher’s Note

All claims expressed in this article are solely those of the authors and do not necessarily represent those of their affiliated organizations, or those of the publisher, the editors and the reviewers. Any product that may be evaluated in this article, or claim that may be made by its manufacturer, is not guaranteed or endorsed by the publisher.
